# Pharmacokinetics, Bioavailability, and Swallowing Safety With Riluzole Oral Film

**DOI:** 10.1002/cpdd.1168

**Published:** 2022-09-27

**Authors:** James Wymer, Stephen Apple, Antoinette Harrison, Bryan Alan Hill

**Affiliations:** ^1^ University of Florida Gainesville Florida USA; ^2^ Mitsubishi Tanabe Pharma America, Inc. Jersey City New Jersey USA

**Keywords:** Riluzole, Amyotrophic Lateral Sclerosis, Dysphagia, Swallow, Neurology, Clinical Trials, Pharmacokinetics and Drug Metabolism, Drug Development, Drug‐food Interactions

## Abstract

Dysphagia is highly prevalent in patients with amyotrophic lateral sclerosis (ALS). Riluzole is a US Food and Drug Administration‐approved treatment for ALS. Riluzole oral film (ROF; Exservan™) contains riluzole in a polymer‐based film matrix. The ROF is administered by placing on the tongue, where it dissolves and the drug is ingested with the saliva. Two clinical trials assessed the safety and tolerability of the ROF. Bioavailability and pharmacokinetics (PK) were evaluated in an open‐label, randomized, single‐dose, replicate crossover study of 50 mg of ROF and riluzole 50‐mg tablets in 32 healthy volunteers. The second study was a videofluoroscopic swallowing examination conducted with nine patients with ALS before and after receiving a single dose of 50 mg of ROF. The primary outcome was change on penetration‐aspiration scale (PAS) scores from pre‐ to post‐dose. Overall, the PK parameters for ROF and riluzole tablets were comparable between treatments and administrations when administered under fasting conditions. Administration of ROF with food resulted in a 15% reduction in area under the curve and a 45% reduction in maximum serum concentration. A total of 44 treatment‐emergent adverse events (AEs) were reported in the study; all were mild in severity. No serious AEs were observed and no subjects discontinued due to AEs. In the swallowing study, very little numerical or categorical change was observed following the dose of ROF. No evidence of deterioration of swallowing function was observed post‐dose. The ROF was bioequivalent to riluzole tablets, was well tolerated, and had no detrimental effect on swallowing.

Amyotrophic lateral sclerosis (ALS) is a progressive and fatal neuromuscular disease characterized by the degeneration of motor neurons in the brain and spinal cord.^1^, ^2^ Patients with ALS typically have a 50% mortality rate within 30 months of symptom onset.^3^ Approximately 25% of newly diagnosed patients with ALS have bulbar‐onset of symptoms, and all patients with ALS experience bulbar symptoms during their disease course.^4^, ^5^ As a result, dysphagia is highly prevalent in patients with ALS.^5^, ^6^ In one study, approximately half of the assessed patients with ALS demonstrated swallowing safety impairments and two‐thirds had impaired swallowing efficiency.^6^ Swallowing safety is therefore an important concern for patients with ALS.

There is no cure for ALS, but 2 therapies have been shown to provide clinical benefit for patients with ALS and have become the standard of care: edaravone and riluzole.^7^, ^8^, ^9^ Riluzole has been associated with survival benefits in ALS.^8^, ^9^ In pharmacokinetic (PK) studies, the tablet formulations of riluzole result in a mean absolute bioavailability of 60%.^10^ The maximal measured plasma concentration (C_max_) and area under the concentration–time curve (AUC) for riluzole were linearly related to dose, and a high‐fat meal significantly reduced the rate and extent of absorption of riluzole.^10^ Riluzole tablet PK parameters were not significantly affected by age or sex, with the exception of having a shorter terminal elimination half‐life (t_1/2_) in elderly subjects.^11^ Metabolism of riluzole occurs predominantly through the cytochrome P450 isoenzyme CYP1A2 to form N‐hydroxyriluzole, with relatively little metabolism via glucuronidation.^12^ A sublingual formulation of riluzole was found to be bioequivalent to the tablet formulation and appeared to be less affected by a high‐fat meal.^13^

Current riluzole administration routes require swallowing a tablet or liquid form, which can be physically challenging and burdensome to patients with ALS. As swallowing becomes more problematic, the current formulations of riluzole may become burdensome for patients with ALS. There is therefore an unmet need for a formulation of riluzole that requires minimal effort in swallowing.

Riluzole oral film (ROF; Exservan™) contains riluzole in a polymer‐based film matrix that is applied to the tongue and rapidly dissolves into the saliva. Once it is dissolved, riluzole in the saliva is then either ingested intentionally or during the normal reflex of swallowing saliva. This report describes the results from 2 clinical studies with the ROF. The first was a PK study of the ROF that was evaluated in healthy subjects (Study 162020) and the second was a swallowing study that assessed the effects of the ROF on swallowing safety in patients with ALS (Study 17MOIR‐0012).

## Methods

The study protocols were approved by institutional review boards for both Study 162020 (Chesapeake Institutional Review Board, Columbia, Maryland) and Study 17MOIR‐0012 (Western Institutional Review Board, Puyallup, Washington). Informed consent was obtained by all study participants. Study 162020 was conducted at the inVentiv clinical facility in Miami, Florida, while Study 17MOIR‐0012 was conducted at the University of Florida in Gainesville, Florida.

### Study 162020

**Figure 1:**
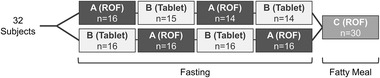
Study 162020 design. ROF, riluzole oral film.

Study 162020 was a single‐center, randomized, single‐dose, open‐label, 5‐period, 3‐treatment, 2‐sequence crossover study, incorporating within the randomization a 4‐period replicate design to evaluate the bioequivalence of ROF 50 mg and riluzole 50 mg tablets (Rilutek) under fasting conditions (overnight, ≥10‐hour fasting period), and incorporating 1 additional treatment (as an additional period, ie, Period 5) to administer ROF 50 mg under fed conditions (Figure 1). This study was conducted in 32 healthy, adult non‐smokers (aged 18–64 years) who had a body mass index of 18.5–29.9 kg/m^2^. On the first day of the first 4 study periods, subjects received either a single dose of ROF 50 mg under fasting conditions without water (Treatment A) or a 50‐mg riluzole tablet with 240 mL of water under fasting conditions (Treatment B) as a 4‐period replicate design. On the first day of the fifth study period, subjects received a single dose of ROF 50 mg administered without water following a fatty meal, as an additional food‐effect arm (Treatment C). In this replicate crossover design, each subject repeated Treatment A and Treatment B for assessment of bioequivalence under fasting conditions (Periods 1–4) and then received Treatment C under fed conditions (Period 5) for a total of 3 treatments in 5 periods by the end of the study. Subjects remained at the research facility from at least 14 hours prior to dosing until collection of the blood sample at 36 hours post‐dose on Day 2 in each study period. Subjects returned to the clinical facility for subsequent collection of blood samples. There was a minimum washout period of 7 days between study periods.

The main assessments in Study 162020 included PK parameters such as AUC from time zero until the last measurable concentration (AUC_0‐t_) or from time zero to infinity (extrapolated; AUC_0‐inf_), the C_max_, the time when C_max_ was observed (t_max_), and the t_1/2_. Blood samples were drawn prior to drug administration (pre‐dose) and at 0.250, 0.500, 0.750, 1.00, 1.50, 2.00, 3.00, 4.00, 6.00, 8.00, 12.0, 16.0, 24.0, 36.0, 48.0, 72.0, 96.0, and 120 hours after dosing in each study period. Efficacy was not assessed in this study.

Plasma samples anticoagulated with EDTA K_2_ were processed with automated protein precipitation and analyzed by high‐performance liquid chromatography with tandem mass spectrometry (AB Sciex Pte. Ltd., Framingham, Massachusetts, API 5000) detection using a Zorbax SB C18, 50 × 4.6‐mm, 3.5‐μm column with milli‐Q water/methanol with ammonium formate and formic acid as mobile phase A and methanol/acetonitrile and formic acid as mobile phase B. The internal reference standards utilized were riluzole‐^13^C‐^15^N_2_ and 2‐amino‐6‐(trifluoromethoxy) benzothiazole. The upper and lower limits of quantitation were 500 ng/mL and 0.5 ng/mL, respectively. All concentration values below the lower limit of quantification were set to zero. The between‐run accuracy and precision had biases ranging from −2.0% to 4.4% and coefficient of variation (CV) of 1.9% to 10.8%, while the within‐run accuracy and precision had biases ranging from −4.7% to 6.0% and CV of 0.8% to 7.2%.

Safety variables that were assessed included adverse events (AEs), vital signs, electrocardiogram, physical examination, and clinical laboratory tests.

### Study 17MOIR‐0012

Study 17MOIR‐0012 was a phase 2 swallowing safety study (NCT03679975) that was conducted in 9 patients with ALS. The study enrolled adult patients (aged 18–80 years) with probable or definite ALS, according to revised El Escorial criteria,^14^ who were on an oral diet and were able to take foods and liquids by mouth. The original plan was to enroll 30 patients for a final sample size of 25 completed patients. With agreement from the FDA, the sponsor decided to terminate the study after 9 completed patients, based on an interim analysis of the first 8 completed patients with no evidence of a harmful effect of one dose of 50 mg of ROF on swallowing function.

The patients participated in a Videofluoroscopic Swallowing Examination (VFSE) with 11 bolus trials and were scored by 2 independent raters on the Penetration Aspiration Scale (PAS). The VFSE is considered the gold standard for studying swallowing and its dysfunction.^15^ The VFSE permits the visualization of bolus flow in relation to structural movement throughout the upper aerodigestive tract in real time.^16^ The VFSE also permits detection of the presence and timing of aspiration.^16^ With the VFSE, clinicians can observe the effects of various bolus volumes, bolus textures, and compensatory strategies on swallowing physiology.^16^

In scoring the PAS, lower PAS scores are better, with a score of 1 or 2 categorized as “safe” and scores of at least 3 as “unsafe” with the following definitions. The category of “safe” includes scores of 1 or 2, with 1 meaning the material does not enter airway and 2 meaning material enters the airway, remains above the vocal folds, and is ejected from airway. The category “penetration” includes scores of 3–5, with 3 meaning the material enters the airway, remains above the vocal folds, and is not ejected from the airway, 4 meaning that the material enters the airway, contacts the vocal folds, and is ejected from the airway, and 5 meaning that the material enters the airway, contacts the vocal folds, and is not ejected from the airway. The final category of “aspiration” includes scores of 6–8, with 6 meaning that material enters the airway, passes below the vocal folds, and is ejected into the larynx or out of the airway, 7 meaning that the material enters the airway, passes below the vocal folds, and is not ejected from the airway despite effort, and 8 meaning that the material enters the airway, passes below the vocal folds, and no effort is made to eject.

The VFSE was conducted and the PAS was scored as previously described.^6^ Patients attended a single clinic visit (Visit 1) where they completed a standardized bolus protocol that included 11 bolus trial presentations in the following order: 3 boluses of 5 mL of thin liquid barium, a regular sip of thin liquid taken from a cup containing 60 mL of thin liquid, consecutive cup sips of remaining thin liquid barium, 3 boluses of a tablespoon of thin‐honey consistency barium, 2 boluses of a tablespoon of pudding consistency barium, and one bolus of a Graham cracker with pudding‐thick barium. Patients were then given a single dose of 50 mg of ROF and the VFSE was repeated at 3 minutes after dosing.

The primary outcome in Study 17MOIR‐0012 was change in PAS scores from pre‐ to post‐dose. The PAS scores were analyzed according to 2 methods: (1) single worst score: the single worst score on any swallowing trial, including determining the number or percentage of patients who stayed the same, increased, or decreased in points; (2) sum of scores: a sum of the 11 PAS scores, including determining the number or percentage of patients who stayed the same, increased, or decreased in points.

In addition, safety and tolerability were assessed through AEs and an oral examination. There were no efficacy assessments conducted in this study.

The ALS Functional Rating Scale‒Revised (ALSFRS‐R),^17^ Functional Oral Intake Scale,^18^ and Eating Assessment Tool 10 (EAT‐10) were used to document the extent of disease and functional status at screening and Visit 1.^19^

## Results

### Study Participants

Healthy adult subjects were enrolled in Study 162020 from February to April 2019. A total of 32 subjects were dosed in the study and 30 completed all treatments (Figure S1). For Study 17MOIR‐0012, 9 patients with ALS were enrolled from May to September 2018 and all 9 completed the study.

### Baseline Demographics and Disease Characteristics

The baseline demographics for both studies and disease characteristics for Study 17MOIR‐0012 are shown in Table 1. In Study 17MOIR‐0012, the median ALSFRS‐R total score of the participants was 38 (range 27–44). Individual patient characteristics in Study 17MOIR‐0012 are listed in Table S1.

**Table 1:** Baseline Demographics and Characteristics for Studies 162020 and 17MOIR‐0012

Baseline Characteristic	Study 162020 N = 32	Study 17MOIR‐0012 N = 9
Age, mean years (SD)	37.6 (10.8)	61.6 (12.0)
Female, n (%)	16 (50)	6 (66.7)
Ethnicity, n (%)		
Not Hispanic or Latino	2 (6.3)	7 (77.8)
Hispanic or Latino	30 (93.8)	2 (22.2)
Height, mean cm (SD)	169.2 (8.0)	167.9 (7.5)
Weight, mean kg (SD)	72.4 (9.5)	77.4 (11.9)
Body mass index, mean kg/m^2^ (SD)	25.3 (2.8)	27.7 (5.6)
ALSFRS‐R total score, median (min, max)	N/A	38 (27, 44)
EAT‐10, median (min, max)	N/A	1 (0, 4)
FOIS score = 7 (normal eating behavior), n (%)	N/A	9 (100)

ALSFRS‐R, ALS Functional Rating Scale‐Revised; EAT‐10, Eating Assessment Tool‐10; FOIS, Functional Oral Intake Scale; N/A, not applicable; SD, standard deviation.

### PK and Bioequivalence in Study 162020

#### Bioequivalence

**Figure 2:**
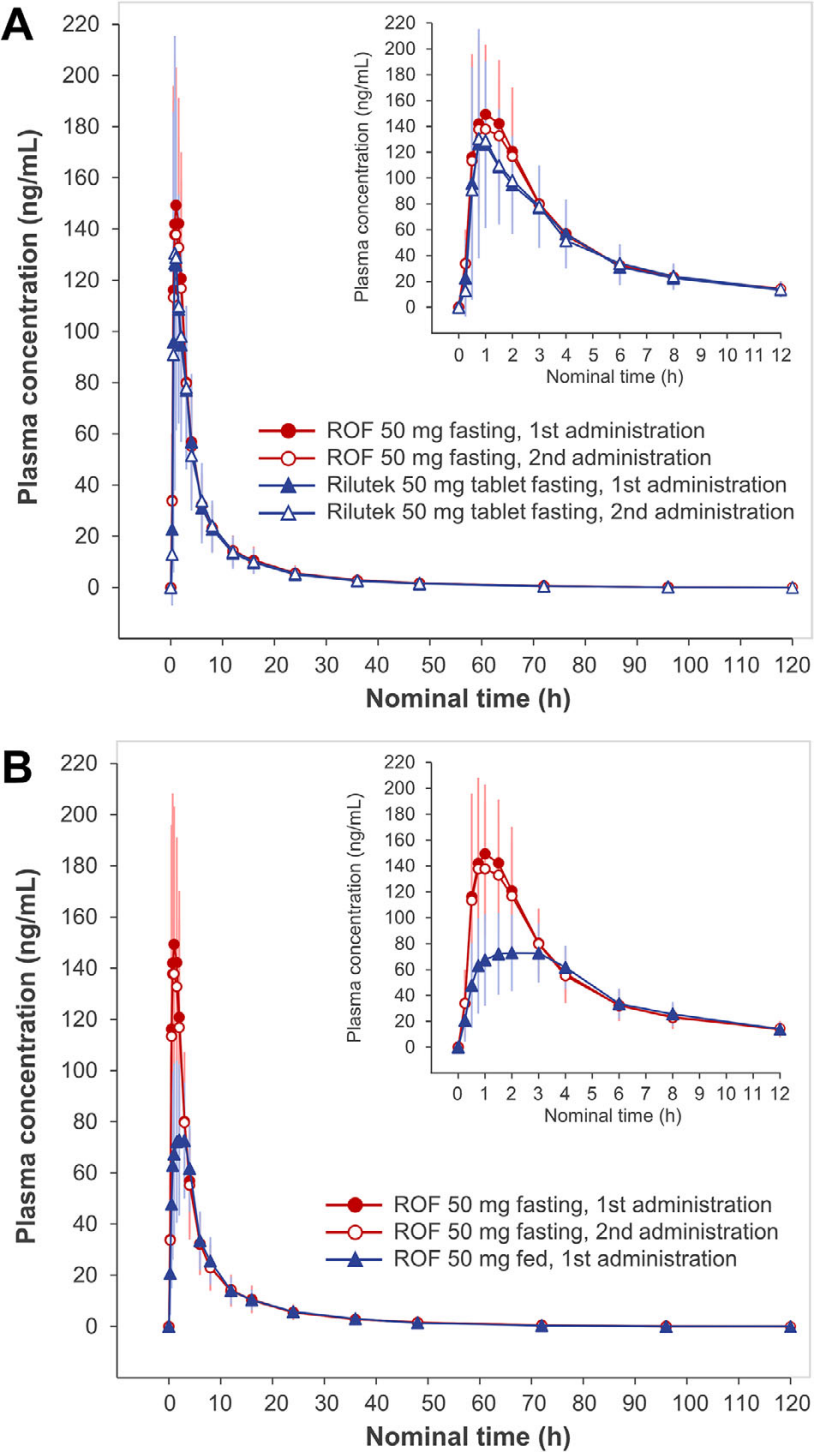
Study 162020 riluzole plasma concentration–time curves (mean ± SD values shown). (A) Treatments A and B, ROF compared with riluzole tablets under fasting conditions. (B) Treatments A and C, ROF under fasting vs high‐fat meal conditions. ROF, riluzole oral film.

The PK parameters for Study 162020 are shown in Table 2. The riluzole plasma concentration–time curves are shown in Figure 2A. The bioequivalence criteria established were met since the geometric mean ratio (A/B) was within the acceptance range of 80.00%–125.00% and the 95% upper confidence bound was lower than 0 (Table 3). Based on these results, it was concluded that ROF 50 mg is bioequivalent to the reference riluzole tablet 50 mg following a 1 × 50 mg dose under fasting conditions (Table 3).

**Table 2:** Study 162020 Pharmacokinetic Parameters

	ROF 50 mg Fasting Conditions (A)		Rilutek 50 mg Tablet Fasting Conditions (B)		
Parameter, Units	1st Administration (n = 31)	2nd Administration (n = 30)	1st Administration (n = 31)	2nd Administration (n = 30)	ROF 50 mg Fed Conditions (C) (n = 30)
AUC_0‐t_, mean ± SD, ng•h/mL (%CV)	832.4 ± 312.8 (37.6)	818.7 ± 298.3 (36.4)	746.6 ± 274.4 (36.8)	768.1 ± 276.1 (35.9)	696.7 ± 223.7 (32.1)
AUC_0‐inf_, mean ± SD, ng•h/mL (%CV)	847.6 ± 315.2 (37.2)	835.1 ± 300.2 (36.0)	761.1 ± 275.9 (36.3)	784.4 ± 278.5 (35.5)	718.8 ± 226.6^a^ (31.5)
Residual area, mean ± SD, % (%CV)	1.9 ± 0.7 (36.5)	2.1 ± 0.8 (36.4)	2.1 ± 0.7 (35.8)	2.2 ± 0.7 (31.6)	2.4 ± 1.1^a^ (46.9)
C_max_, mean ± SD, ng/mL (%CV)	181.0 ± 68.5 (37.8)	169.1 ± 64.6 (38.2)	157.3 ± 72.9 (46.4)	148.2 ± 54.9 (37.1)	92.6 ± 30.8 (33.2)
t_1/2 el_, mean ± SD, h (%CV)	14.1 ± 4.2 (29.7)	15.5 ± 4.5 (28.7)	13.6 ± 4.1 (30.1)	15.0 ± 4.8 (32.2)	13.2 ± 4.2^a^ (31.8)
t_max_, median (min, max), h	1.0 (0.5, 2.0)	1.0 (0.5, 2.0)	1.0 (0.5, 4.0)	0.8 (0.5, 6.0)	1.5 (0.5, 4.1)

AUC, area under the concentration‐time curve; C_max_, maximal measured plasma concentration; CV, coefficient of variation; ROF, riluzole oral film; SD, standard deviation; t½, elimination half‐life; t_max_, time to maximal measured plasma concentration.

^a^ For these values, n = 29.

**Table 3:** Study 162020 Bioequivalence and Food Effect Analyses

Bioequivalence Parameter	CV_WR_	Geometric LSM (A)	Geometric LSM (B)	Ratio (A/B)^a^	Lower CI^b^	Upper CI^b^	95% Upper Confidence Bound^b^
AUC_0‐t_, ng•h/mL	12.7%	780.0	714.5	109.2%	105.7%	112.8%	–
AUC_0‐inf_, ng•h/mL	12.5%	796.0	730.0	109.0%	105.6%	112.6%	–
C_max_, ng/mL	32.7%	–	–	115.8%	–	–	−0.012

Food Effect Parameter	Treatment Comparison	Geometric LSM (C)	Geometric LSM (A)	Ratio^c^	Lower CI^d^	Upper CI^d^	Intra‐subject CV	Inter‐subject CV
AUC_0‐t_, ng•h/mL	ROF fed (C) – ROF fasting (A)	669.8	779.4	85.9%	82.9%	89.1%	9.6%	34.1%
AUC_0‐inf_, ng•h/mL	ROF fed (C) – ROF fasting (A)	679.4	795.4	85.4%	82.5%	88.4%	9.0%	34.0%
C_max_, ng/mL	ROF fed (C) – ROF fasting (A)	90.3	165.5	54.6%	50.4%	59.2%	21.7%	29.9%

AUC, area under the concentration‐time curve; CI, confidence interval; C_max_, maximal measured plasma concentration; CV, coefficient of variation; LSM, least squares mean; ROF, riluzole oral film.

^a^ Point estimate of the geometric mean ratio (A/B).

^b^ Reference‐scaled average bioequivalence approach.

^c^ Calculated using LSM according to the formula: e^(Difference)^ × 100.

^d^ 90% geometric confidence interval using ln‐transformed data.

#### Food Effect

The riluzole plasma concentration–time curves for the food effect comparison are shown in Figure 2B. Similar to what has been shown for the tablet formulation of riluzole, data from the food effect portion of the study involving the administration of ROF 50 mg to healthy volunteers under fasting conditions and with a high‐fat meal indicated decreases of 15% and 45% of AUC and C_max_, respectively, when ROF was administered with food. Moreover, the median time to reach C_max_ was delayed by approximately 0.5 hours (from 1–1.5 hours) when ROF was administered with food (Table 3).

### Swallowing Safety Results

The swallowing study (Study 17MOIR‐0012) included patients with ALS with a broad range of swallowing difficulties. During the pre‐dose assessment, 55.6% of patients were able to swallow safely by PAS standards. Only one patient moved from the “safe” to the “penetration” category of the PAS. No patient had a score indicative of aspiration either pre‐ or post‐dose. Very little numerical or categorical change was observed following the dose of ROF.

Single worst scores: 5 patients (55.6%) had a 1 or 2 as their single worst score and remained the same pre‐ and post‐dose. A sixth patient had a single worst score of 3 both pre‐and post‐dose. One patient had a lower score post‐dose (3 pre‐ and 2 post‐dose), and one patient had a higher score post‐dose (2 pre‐ and 3 post‐dose). The ninth patient had a single score of 5 pre‐dose and a lower score of 3 post‐dose.

**Figure 3:**
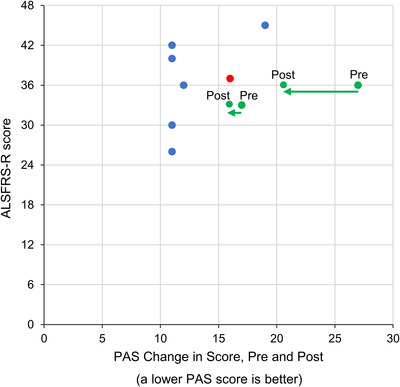
Study 17MOIR‐0012 PAS scores vs ALSFRS‐R score. ALSFRS‐R, ALS Functional Rating Scale‒Revised; PAS, Penetration Aspiration Scale. In the figure, the green circles represent patients who improved in their PAS scores from pre‐ to post‐dose, the red circle represents a patient who had a worsened PAS worst score from pre‐dose to post‐dose, and the blue circles represent patients who had no change in PAS scores from pre‐dose to post‐dose.

Sum of scores: For 7 patients (77.8%), there was no difference between the pre‐ and post‐dose PAS sum of scores. For the remaining 2 patients (22.2%), there was a reduction (ie, an improvement) in PAS sum of scores after dosing of the ROF. No evidence of deterioration of swallowing function was observed post‐dose. There was no correlation between baseline ALSFRS‐R score and changes in PAS scores (Figure 3). Similar findings were obtained for ALSFRS‐R bulbar domain scores vs PAS scores and for EAT‐10 scores vs PAS scores.

### Safety

In Study 162020, a total of 44 treatment‐emergent adverse events (TEAEs) were reported by 19 of the 32 subjects who received at least one dose of the study medication (safety population) (Table 4). The highest frequency of TEAEs was observed in subjects receiving Treatment A. None of these TEAEs were severe or serious, and no trend was observed. No deaths, serious, or significant AEs were reported during this study. The TEAEs reported by >1 subject in any treatment group included oral hypoesthesia (ROF groups) and somnolence (all groups) (Table 4). Oral hypoesthesia was reported at a higher incidence under fasting conditions compared with fed conditions (Table 4).

**Table 4:** Study 162020 Treatment‐emergent Adverse Events

	Statistic	Treatment A ROF 50 mg Fasting Conditions	Treatment B Tablet 50 mg Fasting Conditions	Treatment C ROF 50 mg Fed Conditions	Overall
Number of subjects dosed	n	32	31	30	32
Number of subjects with at least 1 TEAE	n (%)	15 (46.9)	7 (22.6)	8 (26.7)	19 (59.4)
Number of TEAEs	E	23	8	13	44
Number of treatment‐related TEAEs	E	21	4	6	31
Number of serious TEAEs	E	0	0	0	0
Number of subjects who discontinued due to TEAEs	n (%)	0	0	0	0
Number of deaths	n (%)	0	0	0	0
Specific TEAEs					
Hypoesthesia oral	n (%)	12 (37.5)	0	3 (10.0)	14 (43.8)
Somnolence	n (%)	5 (15.6)	5 (16.1)	3 (10.0)	9 (28.1)

E, events; ROF, riluzole oral film; TEAE, treatment‐emergent adverse event.

In Study 17MOIR‐0012, no AEs were reported.

## Discussion

Swallowing can be a considerable challenge for patients with ALS. The ROF was developed as a formulation of riluzole that requires minimal effort in swallowing and is not affected by over‐ or underproduction of saliva, as an alternative for patients with dysphagia. The ROF was well tolerated in both of the clinical studies in this report. The riluzole concentration–time curves, PK data, and bioequivalence assessments from Study 162020 indicated that the ROF was bioequivalent to riluzole tablets under fasting conditions. Riluzole tablets are known to have a food effect, with a 44% reduction in C_max_ when administered with a high‐fat meal.^10^ Consistent with the known food effects with riluzole tablets, the ROF also demonstrated a food effect, with a 15% reduction in AUC and 45% reduction in C_max_ when administered with a high‐fat meal in Study 162020.

Previous swallowing studies have demonstrated that dysphagia is prevalent in patients with ALS, and high proportions of patients with ALS have swallowing profiles that are unsafe (48% of patients tested), inefficient (73% of patients tested), or both (39% of patients tested).^6^ It is therefore important to determine the effects of oral formulations on swallowing in patients with ALS. The results of Study 17MOIR‐0012 indicated that there was no deterioration of swallowing function after the administration of ROF in patients with ALS.

## Conclusions

The ROF represents an alternative delivery system that was bioequivalent to riluzole tablets and was well tolerated in healthy adults. A single dose of 50 mg of ROF had no detrimental effect on swallowing. Together, these 2 studies indicate that ROF represents an alternative administration route in patients with ALS who have dysphagia.

## Author Contributions

James Wymer contributed to study design (Study 17MOIR‐0012), patient enrollment (Study 17MOIR‐0012), writing, clinical interpretation, data interpretation, and approval of final version. Stephen Apple contributed to writing, clinical interpretation, data analysis, data interpretation, and approval of final version. Antoinette Harrison contributed to writing, clinical interpretation, data analysis, data interpretation, and approval of final version. Bryan Alan Hill contributed to writing, clinical interpretation, data analysis, data interpretation, and approval of final version.

## Funding

J.W. has received research funding from Mitsubishi Tanabe Pharma America, Inc. S.A., A.H., and B.A.H. are employees of Mitsubishi Tanabe Pharma America, Inc.

## Supporting information



Supporting Information

## Data Availability

All relevant data are contained within the manuscript.
