# Supplementary material for: Pharmacokinetics, Bioavailability, and Swallowing Safety With Riluzole Oral Film
Source: Clin Pharmacol Drug Dev. 2022 Sep 27;12(1):57–64. doi: 10.1002/cpdd.1168 (PMC10087659; doi:10.1002/cpdd.1168)

**Pharmacokinetics, Bioavailability, and Swallowing Safety With Riluzole Oral Film**

James Wymer^1^; Stephen Apple^2^; Antoinette Harrison^2^; Bryan Alan Hill^2^

1. University of Florida, Gainesville, FL, USA

2. Mitsubishi Tanabe Pharma America, Inc., Jersey City, NJ, USA

**Supplemental Tables and Figures**

**Supplemental Table S1.** Study 17MOIR-0012 Participant Disease Characteristics and PAS Scores at the Time of Study Assessments

Note that due to the inherent variability in ALSFRS-R scores, some of the scores at the time of the swallowing study assessments were slightly different from the baseline values.

| Patient | Age | Sex | BMI | ALSFRS-R Total Score | ALSFRS-R Bulbar Score | EAT-10 | PAS  Pre-dose Score | PAS  Post-dose Score |
| --- | --- | --- | --- | --- | --- | --- | --- | --- |
| 1 | 68 | M | 23.8 | 40 | 11 | 0 | 11 | 11 |
| 2 | 60 | F | 30 | 36 | 12 | 4 | 27 | 21 |
| 3 | 68 | M | 30 | 36 | 12 | 4 | 12 | 12 |
| 4 | 50 | F | 19.4 | 33 | 12 | 0 | 17 | 16 |
| 5 | 67 | M | 22.3 | 45 | 12 | 1 | 19 | 19 |
| 6 | 77 | F | 35.9 | 37 | 12 | 0 | 16 | 16 |
| 7 | 69 | F | 33.7 | 30 | 9 | 2 | 11 | 11 |
| 8 | 37 | F | 31 | 42 | 10 | 4 | 11 | 11 |
| 9 | 58 | F | 29.5 | 26 | 12 | 0 | 11 | 11 |

ALSFRS-R, ALS Functional Rating Scale‒Revised; BMI, body mass index; EAT-10, Eating Assessment Tool-10; F, female; M, male; PAS, Penetration Aspiration Scale.

**Supplemental Figure S1.** Study 162020 Patient Disposition Flow Diagram


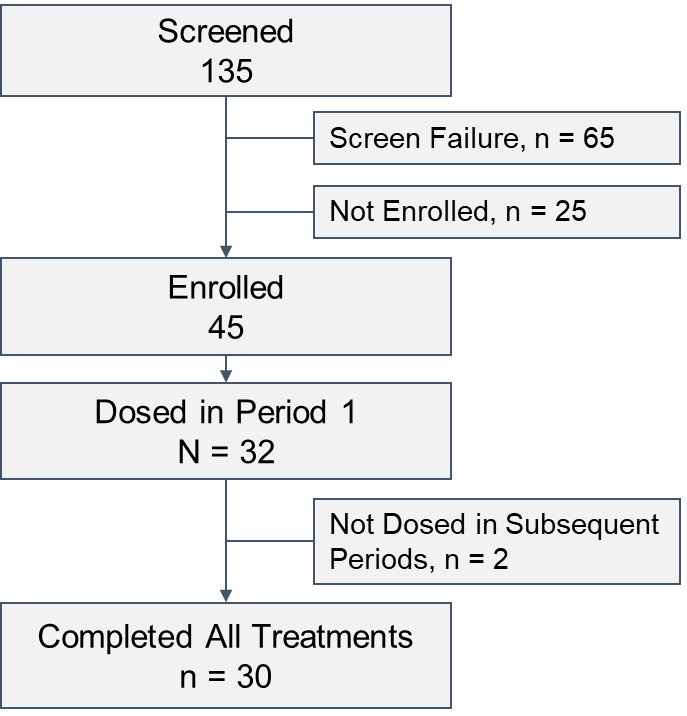

Supplement: Supplementary file 1 — Supporting Information [file CPDD-12-57-s001.docx]
